# Connecting communities: A qualitative investigation of the challenges in delivering a national social prescribing service to reduce loneliness

**DOI:** 10.1111/hsc.12976

**Published:** 2020-03-12

**Authors:** Eleanor Holding, Jill Thompson, Alexis Foster, Annette Haywood

**Affiliations:** ^1^ The School of Health and Related Research (ScHARR) The University of Sheffield Sheffield UK; ^2^ School of Nursing and Midwifery The University of Sheffield Sheffield UK

**Keywords:** community interventions, evaluation, loneliness, qualitative, social isolation, social prescribing

## Abstract

Loneliness is a global public health concern linked to a range of negative health outcomes (Cacioppo & Cacioppo, 2018. *The Lancet. *391(10119), 426). Internationally, this has led to the development of a number of interventions, but these are rarely implemented or evaluated on a large scale. This paper is one of the first of its kind to describe elements of an evaluation of a large‐scale national social prescribing scheme to reduce loneliness, deploying individual link workers to signpost people to community activities. Reporting on findings from interviews with staff (*n* = 25 of which 6 were repeat interviews) and volunteers (*n* = 9) between October 2017 and December 2018 in localities across the United Kingdom. We reflect on the complexities of the link worker role, the challenges of service delivery and the importance of community infrastructure. There was evidence that highly skilled link workers who had developed positive relationships with providers and service‐users were key to the success of the intervention. As well as providing an effective liaison and signposting function, successful link workers tailored the national programme to local need to proactively address specific gaps in existing service provision. For social prescribing services to be successful and sustainable, commissioners must consider additional funding of community infrastructure.

**What is known about the topic?**

- Loneliness is a serious public health issue.
- Social prescribing services are increasingly advocated as a way to reduce pressure on health services and/or improve peoples’ well‐being.
- However, there is a lack of evidence establishing the measureable success, impact or cost‐effectiveness of social prescribing, especially in relation to loneliness.

**What this paper adds?**

- This paper reports on qualitative data from a large‐scale social prescribing intervention to reduce loneliness.
- Link workers with highly developed skills who personalised the national programme to local need were central to the intervention's success.
- Gaps in community infrastructure created challenges for service delivery, highlighting the need for further commissioning of transport, community and befriending services alongside social prescribing.

## 1 INTRODUCTION

Loneliness is increasingly recognised as a global public health concern associated with a range of negative health outcomes (Cacioppo & Cacioppo, 2018; Holt‐Lunstad, Smith, Baker, Harris, & Stephenson, 2015). Comparable in terms of seriousness to obesity and smoking (Hawkley & Cacioppo, 2010), loneliness has been linked to increased risk of coronary heart disease and stroke, depression, cognitive decline and Alzheimer's disease (Valtorta, Kanaan, Gilbody, Ronzi, & Hanratty, 2016). Globally, numerous studies have been undertaken to predict the extent of loneliness with research in the United Kingdom (UK) suggesting that 5% of adults in England report feeling lonely ‘often’ or ‘always’ (Office for National Statistics, 2018), while international reports suggest that one‐third of adults in industrialised countries is affected by loneliness (Cacioppo & Cacioppo, 2018). This highlights the highly subjective and personal nature of loneliness and the difficulty in establishing large‐scale accurate measurements. It is increasingly recognised that loneliness affects people across the life course, not just in older age (Victor et al., 2018). Often linked to social isolation, it is important to recognise a distinction between these terms. Social isolation directly refers to the absence of social contact, whereas loneliness is the experienced feeling of isolation or meaningful contact (Gardiner, Geldenhuys, & Gott, 2018). Thus, it is possible to feel lonely despite a person not appearing isolated and vice versa. The intervention reflected upon in this paper was aimed at loneliness rather than social isolation.

The UK Government set out its vision to address loneliness in its 2018 Strategy, advocating interagency working across sectors, locally developed strategies tailored to individual needs, and increased referral to social prescribing schemes (HM Government, 2018). Social prescribing enables practitioners to signpost service‐users to a range of non‐clinical community activities (South, Higgins, Woodall, & White, 2008). While social prescribing is most frequently drawn upon for dealing with the management of long‐term conditions, it is increasingly promoted as an approach to address loneliness, social isolation and other psychosocial issues (Bickerdike, Booth, Wilson, Farley, & Wright, 2017; Dyson, 2014; Kilgarriff‐Foster & O’Cathain, 2015; NHS Long Term Plan, 2019).

While there are different models of social prescribing (White, 2012), this paper focuses on a service based on the Social Prescribing Network's conceptualisation, where a link worker and a service‐user co‐produce a plan of how to address the service‐user's psychosocial needs by supporting them to access community activities and other services (The Social Prescribing Network, 2018).

Interest in social prescribing reflects broader global awareness of its importance within health policy. For example, in the UK, the Department of Health has called for social prescribing in every locality with a target of over 900,000 people accessing social prescribing schemes (NHS Long Term Plan, 2019). Link workers support people to access a wide range of support, including exercise classes, art therapy, community groups, social services, housing support and befriending services (Chatterjee, Camic, Lockyer, & Thomson, 2018; South & Higgens, 2008). While some studies have reported positive impacts of social prescribing on the adoption of healthier lifestyle behaviours (Mossabir, Morris, Kennedy, Blickem, & Rogers, 2015), self‐esteem (Kilgarriff‐Foster & O’Cathain, 2015) and overall well‐being (Grayer, Cape, Orpwood, Leibowitz, & Buszewicz, 2008), recent reviews have found evaluations to be small scale and limited by poor design and reporting (Bickerdike et al., 2017; Pescheny, Pappas, & Randhawa, 2018). Furthermore, published social prescribing studies have tended to focus on specific health‐related outcomes, drawing on the general health questionnaire (Grayer et al., 2008), the hospital anxiety and depression scale (Mossabir et al., 2015) or the HbA1c for an intervention concerned with type 2 diabetes (Moffat et al., 2019). However, as far as we are aware there are no published studies on social prescribing which focus on loneliness as their primary outcome.

However, a multitude of diverse interventions have been developed to specifically tackle loneliness including gardening schemes, physical activity, befriending, animal interventions and the innovative use of technology (Gardiner et al., 2018; Victor et al., 2018). A number of evidence reviews have assessed the impact of such interventions on loneliness (Cohen‐Mansfield & Perach, 2015; Gardiner et al., 2018; Victor et al., 2018). Despite some interventions demonstrating positive effects, a recent review found that evidence for the effectiveness of interventions on reducing loneliness is limited (Victor et al., 2018). Interventions tend to be smaller scale and focused on older adults, despite loneliness impacting across the life course. There is also little evidence of interventions targeting those most at risk of experiencing loneliness (Victor et al., 2018). Despite this, there is some evidence that interventions adopting a tailored approach (Victor et al., 2018) personalised to the local context (Gardiner et al., 2018) may be more likely to demonstrate positive effects.

In response to this, the British Red Cross (BRC), in collaboration and funded by the Co‐op, developed a national social prescribing programme aimed at supporting people at risk of, or experiencing, loneliness to (re)connect with their communities. The service was rolled out across 37 locations in May 2017. It involves link workers and volunteers working closely with service‐users for up to 12 weeks to develop social links through signposting to community activities and other support. The relatively short‐term nature of this intervention was intentional, with a view to distinguish it from long‐term befriending programmes. The intention was for service‐users to access the programme as an intermediary, or way in, to accessing longer‐term community activities. Each of the 37 areas had one dedicated link worker who were managed by regional managers. The link worker was initially employed by the British Red Cross for 21 hr a week. However, half way through the evaluation this altered with some of the 37 local schemes provided with additional funds to increase link worker hours or employ administrative support. The number of volunteers per locality varied and was reliant on the link workers recruiting local volunteers to the scheme. At the time of evaluation, some schemes had no volunteers while others had 10–15. In total, across the 37 schemes, 390 number of volunteers were registered. A volunteer was required to undergo a compulsory 2‐day training programme which were delivered at two locations across the United Kingdom. The number of hours a volunteer worked was at the discretion of the individual but averaged at between 1 and 3 hr per week. During the evaluation, 5,320 service‐users accessed support through the scheme.

The programme was designed to target those identified as ‘at most risk of loneliness’: young new parents (aged 18–24); individuals with mobility limitations or other health issues; those who recently divorced or separated; people living without children at home and retirees; and the recently bereaved (Kantar Public, 2016), although in practice the programme did not restrict those who supported it. Potential referrers were informed of the service via the link workers. Referral routes included statutory services, NHS, local authorities, the voluntary sector and self‐referral. Link workers were also responsible for advertising the scheme as they saw appropriate for their locality, such as through organised coffee mornings or pop ups at local events. For more information on the scheme, please see the BRC website (https://www.redcross.org.uk/get‐involved/partner‐with‐us/our‐partners/co‐op ) or the Connecting Communities report (Jopling & Howells, 2018).

To understand the impact of the service on loneliness and any barriers to service delivery, the BRC commissioned an independent evaluation. The aim was to understand the impact of the service on service‐user's levels of loneliness using the UCLA loneliness scale and includes analysis from quantitative and qualitative data, alongside a Social Return on Investment and a Matched Comparator analysis. This is described in a subsequent paper (Foster et al., 2020 in preparation).

The data described below forms part of the larger mixed methods evaluation of the social prescribing programme. It focuses on one aspect of the qualitative component of the evaluation and explores the experiences of link workers and volunteers involved in the delivery of the service. To inform future services, the aim of the qualitative component of the evaluation was to understand the challenges of delivering support and the resources and community infrastructure required for successful delivery (this paper), alongside service‐user experiences of receiving support through the programme (to be reported separately).

## 2 METHODS

### 2.1 Sample and recruitment

Semi‐structured telephone interviews with link workers and volunteers were conducted between October 2017 and December 2018. All link workers representing the 37 schemes were initially invited via email to take part in a telephone interview by the University research team. Non‐response was followed up with a phone call before the contact was deleted. As the team did not have permission to contact volunteers directly, an email with the researchers’ University contact details was sent to all volunteers currently supporting service‐users in the scheme through evaluation leads at the BRC. Interested volunteers were asked to contact researchers directly if they wished to participate.

We interviewed all link workers and volunteers who agreed to take part, which amounted to 9 volunteers and 15 link worker interviews. Towards the end of the evaluation (October 2018) at the request of the BRC, we undertook a number of further interviews with link workers to explore new developments and the impact of additional resources that the BRC had provided to the local schemes. During this stage, we undertook six repeat interviews with link workers from the first stage and then purposively sampled an additional four link workers who reflected a particular aspect of our sampling frame in terms of geographical location, size of scheme or additional funds received from the BRC. Interviews were conducted until data saturation.

Copies of the project information sheet and consent form were shared with all participants prior to their involvement in the study and informed consent was taken over the telephone at the time of the interview.

### 2.2 Data collection and analysis

Interviews were semi‐structured and of 30‐ to 90‐min duration. Topic guides were developed which covered link workers’ expectations of the role, training requirements and provision, average working week, experiences of working with service‐users and their perceptions on service implementation, impact and sustainability. With participants’ consent, interviews were digitally recorded, then transcribed verbatim and checked for accuracy before being loaded into NVivo 11 software for coding.

An interpretive thematic analysis approach was used following the principles of open coding, followed by more detailed selective coding (Bryman, 2012; Seale, 2004). Once a number of transcripts had been received, these were read separately by three members of the research team (EH, JT and AH) before initial coding frameworks for the two interview groups were developed. The coding frameworks were continually refined. The research team met regularly to discuss any disagreements before final coding frameworks were agreed and applied to the transcripts (EH and JT). Constant comparison, combining simultaneous coding and analysis of the data, was used to ensure the validity of the coding frameworks (Taylor & Bogdan, 1998).

Ethical approval for this project was granted by the School of Health and Related Research (ScHARR), University of Sheffield's ethics committee (015364).

## 3 FINDINGS

In all, 6 male and 13 female link workers, alongside 4 male and 5 female volunteers were interviewed. The scheme was a new intervention and had been running for 1 year at the time of the interviews. Therefore, the majority of link workers had been in post for less than a year, with 7 being in post for the duration of the programme. Only one volunteer had been in post for less 6 months, with the majority volunteering for 6–10 months. The professional backgrounds of both link workers and volunteers varied, with the majority having had previous roles in healthcare services (*n* = 6) and third sector organisations (*n* = 6). Other professional backgrounds included secondary/higher education (*n* = 4), private sector business (*n* = 3), other public sector organisations (*n* = 2), emergency services (*n* = 2), travel and tourism (*n* = 1) and administrative services (*n* = 2). Two volunteers had been full‐time carers for over 10 years.

Interviewee's accounts of service implementation and delivery revealed challenges, which some link workers sought to address through innovative practices. Three key themes arose relating to the delivery of the service: (a) the complexity of the link worker role and the importance of their individual attributes, and relationships, to the success of the intervention, (b) challenges to service delivery and (c) issues of existing (or lack of existing) community infrastructure.

### 3.1 The link worker role

The original programme specification suggested that typically once referred to the service, service‐users will be visited by their link worker and together they set goals for the 12‐week programme. They will then be matched with a volunteer who signposts and accompanies the service‐user to local community activities which align with their goals. Progress against these goals is monitored through the link worker via phone calls and joint meetings with the volunteer over the 12‐week period. Therefore, the volunteer role has greater emphasis on developing the 1:1 relationship with the service‐user, while the link worker has more of an oversight of the overall programme in their local area.

However, the interviews revealed clear differences in interpretation and undertaking of the link worker role. Core components of the role included: assessing referrals and managing service‐user caseloads, sourcing community activities for onward signposting, setting goals with service‐users, matching up volunteers with service‐users, managing the volunteers and monitoring and recording of statistics and administrative tasks. At the time of the initial evaluation interviews, many local schemes had not recruited any/sufficient volunteers to support service‐users because the service was in its infancy. This meant that some link workers were undertaking both their role and that of the volunteer. Inevitably, this led to some link workers feeling overwhelmed by the workload. This was a particular issue during the first round of interviews, before the BRC invested further funding into the programme:

It’s very challenging, I’ll be honest, yes it’s really tough… I love the work but the hours and everything, it’s really challenging. It does feel like a full‐time job in 21 hours…with more hours it could be a very enjoyable job. But as it is at the moment, yes it is very hard and very challenging because I’m by myself. So why am I doing this, you know, the volunteers, and the people that walk in, that’s what, kind of keeps me here. (LW7, first year)

However, even following volunteer recruitment, many link workers continued to undertake more of a hands‐on approach with the service‐users than was initially envisaged for the role, often because they had already developed relationships with the service‐user or because the hours that the volunteer could give did not fit in with the service‐user's requirements.

Due to the complex needs and requirements of their service‐users, many link workers found the role could be emotionally and physically demanding:

It does come with challenges sometimes because it’s not just going out with a service‐user. I can’t just say it’s going to take me an hour. It could take longer than that because it’s life, you are going to see real people. So it comes with challenges. (LW17, follow up)

The ‘success’ of each local scheme was a complex issue and dependant on, among many other things, (a) receiving sufficient service‐user referrals into the scheme; (b) having sufficient activities, opportunities and networks to signpost the service‐user to and (c) having enough link worker and volunteer hours to ensure the scheme could run. Given that this was a new scheme, it was the link workers’ role to undertake sufficient networking with local services to embed the service within the local area. Those schemes that appeared to have larger numbers of service‐users both in and signposted out seemed to be driven by a link worker with particular attributes. These included being proactive, empathetic, having time to dedicate to the service‐user, not making assumptions regarding the activities that people wanted and ensuring that the service was personalised and tailored to the needs of the local area.

The person‐centred approach of the scheme was viewed extremely positively by all link workers:

…I think putting the onus onto people and saying, well you know, this is about you. Support is for you. It’s what you want to achieve from it. It’s probably a brilliant refreshing concept, but I think it’s quite new to the members of the public. (LW10, follow up)

However, many link workers felt that the person‐centred ethos could be constrained by the 12‐week time limit on service provision. So while the link worker had some autonomy in how they rolled out the service locally, these were still bound within the overall service specification.

### 3.2 Challenges to service delivery

As with any new service, interviewees reported challenges to service delivery, including the need for longer‐term support for some service‐users, the suitability of referrals and a lack of community resources and infrastructure. Link workers utilised a number of strategies to combat these challenges, inevitably leading to different variations of the service model being adapted to local needs.

### 3.3 The need for longer‐term support

As the service was only intended as short‐term signposting, most link workers and volunteers tried not to exceed the 12‐week support period stipulated in the service specification. However, they felt that for some service‐users longer‐term support was necessary due to the complex nature of loneliness. This demonstrates the importance of services being flexible in their delivery models to enable workers to tailor support to the service‐users’ needs, and highlights that some people need more than a short‐term signposting service:

So, there’s loads of new studies and things that are being done all the time about loneliness but I read something that said approaches need to be on a longer scale moving forward… And so, putting that person at the centre, again. Not trying to find a programme that’s going to fit everybody but, looking at what’s helpful to that person. And I really agree with that, because I think loneliness is so complex and because you’ve got the influences of what it can do to your physical and mental health. I don’t really think that 12 weeks is long enough for some people. (LW7, first year)

Providing longer support was particularly relevant for service‐users who had not been successfully signposted into community activities (e.g. due to mobility/health issues) during the 12‐week period. However, a small number of link workers felt that the length of intervention was sufficient, particularly to manage expectations and to avoid attachment, which was described by some as emotionally difficult:

I think the hardest thing is the separation at the end of twelve weeks. (LW1, first year)

At the very beginning we were taking service‐users up to eleven to twelve weeks I will be honest… And what I noticed was the relationship was getting a bit too close for comfort. In terms of it becomes kind of an attachment then. Service‐users want to cling on… But I find now what we’ve done is we are kind of quite strict on how we go in. (LW18, follow up)

### 3.4 Inappropriate referrals and challenges to signposting

Despite reiterating the services remit to potential referrers, some link workers felt that they still received inappropriate referrals (such as those who needed longer‐term support or had complex needs) from mental health services and social services. Some link workers suggested that this may be a result of over stretched statutory services, suggesting that the intervention was filling a gap in service provision. Link workers wanted to support these vulnerable service‐users, but this resulted in them providing more specialist support than was initially anticipated, acting as unqualified social or advocacy workers for service‐users (e.g. assisting them with benefit claims, legal issues and housing forms):

The barriers is basically we are lurching towards being case workers…or at least in my job, in what I do, you know, a lot of what I do I consider case work…Nobody else is doing it. It’s filling a gap left by statutory services really. (LW6, first year)

We do get quite an assortment of inappropriate referrals, referrals where it’s just a load of people actually needing more support. It’s more like a kind of social work, the thing that they’re needing. (LW6, first year)

Link workers and volunteers also discussed the difficulty in supporting individuals with severe mental and/or physical health problems, which limited their ability to engage with community activities:

We’re getting too many referrals from the elderly…. the two ladies that I’ve been allocated as part of my [role] are elderly and that’s fine… but the end game, the 12 weeks where we’re trying to get them to connect with their own community and get back into it again… something happens to them health‐wise and we’ve no control over that. (V6)

There’s a lot of referrals coming which are more respite care which is not within the remit…and people that you would never, ever give a conclusion for in any case, because they can’t get out in any case because they are wheelchair bound (V8)

### 3.5 The importance of the community infrastructure

Social prescribing programmes are largely dependent on the infrastructure of local communities including availability of community activities and transport. The availability of services varied greatly across localities. For example, a link worker operating in an affluent area in the South of England discussed the abundance of community activities in their local area:

There’s groups for everything, you know. Everything I thought of which would make an idea for a group, there's a group out there…Scrabble groups, reading groups, there's walk and talk groups, there's cycling groups, there's coffee mornings galore. (LW12, first year)

While other link workers reported that in their specific geographical areas, there was a lack of services to signpost service‐users to, which imposed difficulties for the programme:

So yeah, we kind of signpost if it’s appropriate. Sometimes it’s difficult to signpost because services aren’t available. At the time when you find a service, it’s available to signpost. When you make contact with that service, ready to signpost, it’s not there. (LW17, follow up)

Some link workers utilised innovative strategies to address gaps in provision. For example, two particularly successful services in terms of the number of referrals received and signposts made, developed self‐sustaining groups around their service‐users’ interests. These included a walking group in one scheme, a model railway group in another scheme and a choir for young people for a third scheme that recognised the underrepresentation of this group within their service. Another service operating in a large rural area set up group sessions in the local community hall for both service‐users and the wider community:

I mean we have got ten service‐users in the model railway group… And that’s beginning to self‐manage itself already. And I knew there was somebody who was interested in model railways and I had somebody who was interested in history and somebody else that was interested in modelling. So you sort of say, well how can you get three people together and we said well, how about making a model of a live railway station from the 1950s? And they all said yes, that might be interesting. And we brought three of them together and then somebody came in off the street and said I’ve heard about this, can I join the group? And we’ve now got ten who are attending and it’s just fantastic. (LW15, follow up)

Many service‐users who had not been successfully signposted required referral to longer‐term befriending programmes but in the majority of cases these programmes did not exist locally or there were long waiting lists. Some link workers and volunteers believed adding a befriending element to the service could address this unmet need. However, the resource implications of this were acknowledged:

Although I know it would open a whole new can of worms in terms of the resources that we would need to feed in to the service…But I feel like it would be putting the service‐users first by being able to provide that slightly longer‐term support for people where there is a gap in befriending services. (LW4, first year)

Furthermore, there were challenges supporting service‐users with mobility/health issues to maintain engagement in activities because of difficulties utilising transport. This was amplified in cases where the service‐user had relied on the link worker or volunteer for transport to and from activities during the 12‐week programme.

Some link workers had been successful in sourcing sustainable transport for service‐users so they could continue to attend activities at the end of the intervention. However, community transport varied across localities, again reflecting the importance of local infrastructure for the success of social prescribing services. When access to community transport is not available, this can incur a financial burden on service‐users:

I think [transport] is really important because I think that’s the one thing that let’s our service‐users down. Without transport, they are not able to get out and about… And it may be just the one class they want to get to, having that vehicle or having somebody being able to take them on a weekly basis, is consistency…consistency is really, really important. (LW17, follow up)

## 4 DISCUSSION

This study forms part of the first large‐scale evaluation of a national social prescribing intervention to alleviate loneliness. Previous evaluations of social prescribing interventions have been small scale, focusing on one locally developed service.

The aim of this qualitative component was to understand the challenges of delivering the programme and the resources and community infrastructure required for its successful delivery. What emerged was how a national programme had been developed according to the local context.

Our findings highlight a number of challenges to programme delivery: (a) the need for longer‐term support, (b) inappropriate referrals and (c) lack of community infrastructure. Despite the 12‐week support period being sufficient for some service‐users, many link workers felt that the chronic nature of loneliness required longer‐term support in some cases. In common with Moffatt, Steer, Penn, and Lawson (2017), inappropriate referrals meant that some service‐users with severe mental and physical problems were referred to the service, which limited the link workers/volunteers abilities to effectively support them.

It is clear that community infrastructure is pivotal in the success of social prescribing services (Whitelaw et al., 2017). In this study, transport was a key factor in vulnerable service‐users continuing the activities they had been signposted to. Similar to other evaluations of locally developed social prescribing interventions (Dayson, Bashir, & Pearson, 2013), sourcing sustainable transport was a major barrier to accessing services due to the varying availability of transport across localities. Similarly, our findings indicate that link workers are constrained by the lack, or high turnover, of external services.

Since the coalition government of 2010, the UK’s budget has been dominated by cuts in funding and public services as part of the ongoing era of ‘austerity’ (Harris, Springett, Mathews, Weston, & Foster, 2019; Lowndes & Pratchett, 2012). Such cuts have disproportionately affected areas of high deprivation and levels of need (Lowndes & Pratchett, 2012), meaning that the poorest and most vulnerable have been the hardest hit (Hastings, Bailey, Gramley, Gannon, & Watkins, 2015). To manage reduced budgets alongside the increased prevalence of complex needs, new models of care are being developed, such as social prescribing, to manage demand (NHS Long Term Plan, 2019). However, as is clear in the present study, the sustainability of such programmes is dependent on the availability of community infrastructure. Funding for the voluntary and community sector has been disproportionately hit by the austerity era, leading to increased demand for reduced services (Jones, Meegan, Kennett, & Croft, 2016). Link workers in this study encountered difficulties sourcing befriending, transport and other community services. At times, they also had to act as case workers for service‐users with complex needs who had fallen through the gap of other over stretched services. Cuts in public spending and services may therefore present a threat to the future sustainability of the service, and social prescribing more widely.

Link workers utilised a number of strategies to combat gaps in service provision, leading to variations of the national model being tailored to local need. Areas with the most ‘success’, in terms of the numbers of service‐users enrolled in the programme, were spearheaded by an innovative link worker with highly developed skills and relationships both with external organisations and their service‐users. Such workers demonstrated genuine passion and drive, fostered through previous community‐based roles, and utilised innovative strategies to address gaps in community infrastructure. Where resources did not exist locally, link workers built on the concept of peer support (Harris et al., 2015) by bringing together service‐users with common interests to develop and run their own self‐sustaining groups. Wildman, Valtorta, Moffatt, and Hanratty (2019) found that sustainable projects in areas of high deprivation were tailored to their individual context, harnessed the assets of the local people and were developed in co‐creation around service‐user's wants and needs. Similarly in this study, the areas that sustained support for the most service‐users had tailored the programme and coproduced activities according to local infrastructure and needs. In common with other research into the importance of the link worker role in developing relationships, engaging service‐users and ultimately improving outcomes (Keenaghan, Sweeney, & McGowan, 2012; Moffatt et al., 2017; Whitelaw et al., 2017), link workers in this study appear to be the ‘*key feature to the success of social prescribing*’ (Bickerdike et al., 2017:15).

### 4.1 Implications for practice and policy

There is evidence that social prescribing can have a positive impact on the health and well‐being of service‐users (Bickerdike et al., 2017). Some evaluations of locally developed services have also found more general links between social prescribing and improvements in isolation and loneliness (Woodall et al., 2018). However, policy decisions to implement social prescribing services often fail to recognise the lack of capacity and limited resource within the community and voluntary sector (Harris et al., 2019).

Some link workers in this study encountered difficulties accessing external services. In particular, workers discussed the increased demand for befriending and transport services for the alleviation of loneliness. For social prescribing services to be successful and sustainable, commissioners must consider the further funding of onward activities in addition to the link worker element (Dayson, Bashir, Bennett, & Sanderson, 2016). If this is not possible, organisations must cultivate a strong workforce able to develop innovative activities according to the local context. Organisations also need to be mindful of the challenges of the role and develop structures to support staff. This was demonstrated by the BRC in this programme, who invested in additional staff to increase capacity and help manage workloads.

### 4.2 Strengths and limitations

The national scale of the evaluation, involving multiple locally delivered schemes is a clear strength of this paper. It draws worthy attention to the challenges of delivering this type of intervention which is important for future development of social prescribing services.

Data were collected over a 2‐year period with link workers and volunteers, enabling us to gain longitudinal perspectives on the delivery and experiences of the programme. In some cases, repeat interviews were undertaken with link workers to enable follow‐up and reflection. However, a clear limitation of this study is its lack of evidence on the social patterning of loneliness across social class, poverty and deprivation. This is an omission given the findings of the importance of community infrastructure which has been disproportionally affected by funding cuts across geographical areas. Case study analysis could have also provided a more robust comparison of approaches across the different areas. Both issues need further exploration in future mixed methods studies.

## 5 CONCLUSION

Given the increased investment and policy interest in social prescribing services, a body of evidence is emerging on its effectiveness. What this study adds is an understanding of the complexity of delivering large‐scale social prescribing through locally tailored schemes. It is clear that link workers with highly developed skills who are able to tailor the programme to the needs of the local context are key to the success of the intervention. A number of challenges emerged including the availability of transport, befriending and other community services. Social prescribing services seeking a positive impact on loneliness need to consider the availability of resources and transport infrastructure. Further large‐scale evaluations of social prescribing and similar interventions on loneliness are required given the current lack of evidence on their effectiveness (Husk, Elston, Gradinger, Callaghan, & Asthana, 2019; Victor et al., 2018).

## CONFLICT OF INTEREST

The authors declare no conflict of interest.
